# Determining the QRS axis: visual estimation is equal to calculation

**DOI:** 10.1007/s00399-025-01065-x

**Published:** 2025-01-27

**Authors:** Johanna Mueller-Leisse, Giulia Syrbius, Henrike Aenne Katrin Hillmann, Joerg Eiringhaus, Stephan Hohmann, Christos Zormpas, Nizar Karfoul, David Duncker, Christian Veltmann

**Affiliations:** 1 Hannover Heart Rhythm Center, Department of Cardiology & Angiology, Hannover Medical School, Carl-Neuberg Str. 1, 30625 Hannover, Germany; 2 Heart Center Bremen, Electrophysiology Bremen, Bremen, Germany

**Keywords:** 12-Lead-ECG, Outflow tract PVCs, Hexaxial reference system, ECG basics, PVC morphology, 12-Kanal-EKG, Ausflusstrakt-VES, Crabrera-Kreis, EKG-Grundlagen, VES-Morphologie

## Abstract

**Background:**

The QRS axis of the electrocardiogram (ECG) is often considered in clinical practice, but its determination is frequently limited to a rough estimation, such as “normal”, with left or right deviation, and superior or inferior in the case of premature ventricular complexes (PVCs). However, a more exact determination of the QRS axis may be warranted in certain scenarios, such as to determine the origin of PVCs more precisely, and is attainable by visual estimation using the hexaxial reference system.

**Objectives:**

The aim of this study was to determine how well such an estimation of the QRS axis would correlate with the axis calculated by formulas.

**Materials and methods:**

A PVC database from 2012–2020 was used to extract 12-lead ECGs of patients with outflow tract PVCs and analyze the QRS axes of regularly conducted beats as well as PVCs. QRS axes were determined visually by two physicians with the help of the hexaxial reference system to an accuracy of 10° on the one hand, and were calculated using three previously described formulas based on QRS voltages on the other.

**Results:**

A total of 216 QRS complexes from 108 patients were analyzed (108 regularly conducted beats and 108 PVCs). Estimated QRS axes of regularly conducted beats and PVCs were 39 ± 40° and 88 ± 15°, respectively. Calculated QRS axes of regularly conducted beats according to the three formulas were 37 ± 40°, 36 ± 40° and 35 ± 38°, respectively. Calculated QRS axes of PVCs according to the three formulas were 87 ± 15°, 87 ± 14° and 86 ± 16°, respectively. Correlation coefficients showed strong correlations between the estimated and the three calculated values for regularly conducted beats (0.98, 0.97 and 0.98) and PVCs (0.94, 0.94 and 0.94).

**Conclusions:**

A sophisticated visual estimation of the QRS axis correlates well with voltage-based calculations and can therefore be considered sufficient for most purposes.

## Abstract (de)

**Hintergrund:**

Die QRS-Achse des Elektrokardiogramms (EKG) wird in der klinischen Praxis oft berücksichtigt, häufig aber nur annähernd bestimmt und in „normal“, überdreht links oder überdreht rechts unterteilt. Im Falle von ventrikulären Extrasystolen (VES) wird häufig lediglich zwischen inferiorer und superiorer Achse unterschieden. In bestimmten Situationen, wie der genaueren Bestimmung des Ursprungsorts von VES, kann jedoch eine präzisere Bestimmung der QRS-Achse sinnvoll sein. Dies ist durch visuelle Beurteilung unter Nutzung des Cabrera-Kreises möglich.

**Ziel der Arbeit:**

Ziel dieser Arbeit war es zu prüfen, inwieweit eine unter Nutzung des Cabrera-Kreises visuell bestimmte QRS-Achse mit der durch Formeln berechneten QRS-Achse übereinstimmt.

**Material und Methoden:**

Aus einer von 2012 bis 2020 geführten VES-Datenbank wurden 12-Kanal-EKGs von Patienten mit monomorphen VES mit Ausflusstrakt-Morphologie extrahiert. Zwei Ärzte analysierten die QRS-Komplexe der normal übergeleiteten Schläge sowie der VES. Hierbei wurden die QRS-Achsen einerseits visuell mithilfe des Cabrera-Kreises bestimmt (Genauigkeit von 10°) und andererseits mithilfe dreier beschriebener QRS-Voltage-basierter Formeln berechnet.

**Ergebnisse:**

Es wurden 216 QRS-Komplexe von 108 Patienten analysiert (108 normal übergeleitete Schläge und 108 VES). Die visuell bestimmte QRS-Achse der normal übergeleiteten Schläge betrug im Mittel 39 ± 40°, die der VES 88 ± 15°. Die anhand dreier Formeln berechneten QRS-Achsen der normal übergeleiteten Schläge betrugen 37 ± 40°, 36 ± 40° und 35 ± 38°, die der VES 87 ± 15°, 87 ± 14° und 86 ± 16°. Die Korrelationskoeffizienten zeigten eine gute Korrelation zwischen dem visuell bestimmten Wert und den drei berechneten Werten für die QRS-Achse von normal übergeleiteten Schlägen (0,98, 0,97 und 0,98) und von VES (0,94, 0,94 und 0,94).

**Schlussfolgerung:**

Eine genauere visuelle Bestimmung der QRS-Achse mithilfe des Cabrera-Kreises korreliert gut mit voltagebasierten Berechnungen und weist somit für die meisten Fragestellungen eine ausreichende Genauigkeit auf.

## Introduction

The electrocardiogram (ECG) is a crucial basic tool in the diagnostics of arrhythmias. Determination of the QRS axis forms part of the analysis and plays a role in many clinical questions including the recognition of right of left heart strain, conduction system disturbances, acquired or congenital heart disease, and the identification and localization of arrhythmias [1–8].

In clinical practice, a rough visual estimation of the QRS axis is often made to differentiate an inferior versus superior axis or identify overt right or left axis deviation [6, 9–12].

However, a more exact assessment of QRS axis in degrees may be of interest in some cases, including in the determination of the focus of premature ventricular complexes (PVCs) [13–20]. It can be achieved by performing calculations or plotting on a graph using different limb leads. Although there is uncertainty which leads should best be used because of different weighting of the bipolar and unipolar leads, these differences seem negligible for most clinical questions [21, 22]. QRS axis can also be assessed much more easily by visual estimation to an accuracy of 10° with the help of the hexaxial reference system [23]. The authors sought to investigate how well this estimation of the QRS axis, including that of PVCs, correlates with calculations using different formulas.

## Methods

**Fig. 1:**
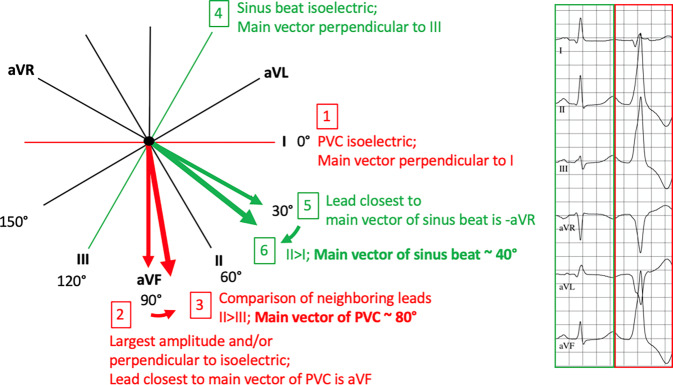
Example of an electrocardiogram and demonstration of steps to estimate the QRS axis of the PVC (steps 1–3) and the sinus beat (4–6) using the hexaxial reference system. (*PVC* premature ventricular complex)

A PVC database obtained at the authors’ center between 2012 and 2020 from patients with predominantly monomorphic PVCs and outflow tract appearance was used to extract 12-lead ECGs. Patients with a superior PVC axis and with a stimulated ventricular rhythm were excluded. QRS axes were determined visually by two physicians with the help of the hexaxial reference system to an accuracy of 10° on the one hand (Fig. 1), and were additionally calculated with the help of three formulas using manually measured QRS voltages on the other: 1) ± Arctan ((2 × aVF)/(Sqr(3) × I)), 2) ± Arctan ((I + 2 × III)/(Sqr(3) × I)) and 3) ± Arctan (aVF/I) [17, 22, 24–27]. In cases where the two visual estimations diverged ≥ 10°, ECGs were reviewed to find the accurate solution.

Statistical analysis was performed with SPSS version 28 using Pearson correlation and the Friedman test for continuous non-parametric dependent variables with Bonferroni correction. Continuous variables are presented as mean ± standard deviation. A *p*-value < 0.05 was considered statistically significant.

The study complied with the Declaration of Helsinki and was approved by the local ethics committee. All participants provided written informed consent.

## Results

The study population consisted of 108 patients (53% male) with a mean age of 54 ± 17 years. Baseline characteristics are shown in Table 1. A total of 216 QRS complexes were analyzed (108 normally conducted beats and 108 PVCs). Mean QRS durations were 101 ± 21 and 155 ± 24 ms for normally conducted beats and PVCs, respectively. Estimated QRS axes using the hexaxial reference system were 39 ± 40° and 88 ± 15° for normally conducted beats and PVCs, respectively. Calculated QRS axes of normally conducted beats according the formulas 1, 2 and 3 were 37 ± 40°, 36 ± 40° and 35 ± 38°. Calculated QRS axes of PVCs according to the formulas 1, 2 and 3 were 87 ± 15°, 87 ± 14° and 86 ± 16°. Correlation coefficients showed strong correlation between the estimated and the three calculated QRS axes for normally conducted beats (0.98, 0.97, 0.98; *p* < 0.001) and for PVCs (0.94, 0.94 and 0.94; *p* < 0.001). Correlation coefficients between formulas were all ≥ 0.99; *p* < 0.001 (Table 2).

**Table 1:** Patient and ECG characteristics

Patient and ECG characteristics (*n* = 108)	
Patient characteristics	
*Male gender, n (%)*	58 (53)
*Mean age, years*	54 ± 17
*Mean BMI, kg/m*^*2*^	27 ± 5
*Mean LVEF, %*	48 ± 13
*Comorbidities, n (%)*	
Heart failure	56 (51)
Coronary heart disease	19 (17)
Valvular heart disease	5 (5)
Renal disease	7 (6)
Diabetes	11 (10)
Arterial hypertension	59 (55)
Atrial fibrillation	11 (10)
*Antiarrhythmic drugs, n (%)*	
Beta-blocker	67 (61)
Class I	3 (3)
Class III	1 (1)
ECG	
*Mean PVC count on 10‑s strip*	4 ± 2
*Bigeminus, n (%)*	22 (20)
*QRS duration, ms*	101 ± 21
*QRS duration (PVC), ms*	155 ± 24
*RS transition*	4.2 ± 1
*RS transition (PVC)*	2.8 ± 1
*QRS axis, estimated, °*	39 ± 40
*QRS axis (PVC), estimated, °*	88 ± 15

*BMI* Body mass index, *ECG* Electrocardiogram, *LVEF* Left ventricular ejection fraction, *PVC* Premature ventricular contraction

**Table 2:** Estimated vs. calculated QRS axis

Estimated vs. calculated QRS axis			
	Mean values, °	Mean difference, ° (*p*-values)	Correlation coefficient (*p*-values)
*Sinus axis, estimated vs. calculated; n* *=* *108*			
Estimated	39 ± 40	–	–
Formula 1	37 ± 40	+2.4 ± 8 (0.623)	0.98 (< 0.001)
Formula 2	36 ± 40	+3.5 ± 9 (0.004)	0.97 (< 0.001)
Formula 3	35 ± 38	+4.2 ± 9 (< 0.001)	0.98 (< 0.001)
*PVC axis, estimated vs. calculated; n* *=* *108*			
Estimated	88 ± 15	–	–
Formula 1	87 ± 15	+1.7 ± 7 (0.002)	0.94 (< 0.001)
Formula 2	87 ± 14	+1.2 ± 7 (1.000)	0.94 (< 0.001)
Formula 3	86 ± 16	+2.1 ± 7 (0.023)	0.94 (< 0.001)

*PVC* Premature ventricular contraction

Estimated axes tended to have greater values compared to calculated axes. Mean deviation of the estimated axes from the calculated axes using formulas 1, 2 and 3 of regularly conducted beats were +2.4 ± 8° (*p* = 0.623), +3.5 ± 9° (*p* = 0.004) and +4.2 ± 9° (*p* < 0.001). Mean deviation of the estimated axes from the calculated axes of PVCs were +1.7 ± 7° (*p* = 0.002), +1.2 ± 7° (*p* = 1.0) and +2.1 ± 7° (*p* = 0.023).

The mean deviations between formulas 1, 2 and 3 were as follows. Normally conducted beats: +1.1 ± 6° (formula 1 vs. 2; *p* = 0.492), +0.7 ± 7 (formula 2 vs. 3; *p* = 0.009) and +1.8 ± 3 (formula 1 vs. 3; *p* < 0.001); PVCs: −0.5 ± 1° (formula 1 vs. 2; *p* = 0.004), +0.8 ± 2 (formula 2 vs. 3; *p* = 0.027) and +0.3 ± 2 (formula 1 vs. 3; *p* = 1.0).

## Discussion

Although determination of the QRS axis remains a rough estimate in clinical practice, an estimation to an accuracy of 10° with the help of the hexaxial reference system, without the need for calculations or plottings, has been described [23]. The authors sought to validate this method by comparison with calculations, with a focus on idiopathic PVCs, as a more accurate determination of the PVC axis may help in differentiating between different PVC foci [18]. Their data confirm a strong correlation between estimated QRS axes and calculated axes using three previously described voltage-based formulas involving leads I, II and aVF [22, 24]. Correlation was strong for both normally conducted beats and PVCs using any of the formulas. As expected, regularly conducted beats showed much greater variation in QRS axis compared to PVCs, which were of outflow tract morphology. Small differences between estimated and calculated values as well as between the three applied formulas did reach statistical significance, but these differences were < 10° and thus are not of clinical significance. The data therefore confirm that an estimation of the QRS axis of both regularly conducted beats and PVCs of outflow tract origin to an accuracy of 10° is reliable, eliminating the need for plotting or calculations.

## Limitations

A limitation of the study is that QRS axes were not calculated with areas but amplitudes, which is less accurate. However, using amplitudes is still an accepted equivalent. Another limitation is that visual estimation of the QRS axis may be considered non-objective. However, this reflects clinical practice. Moreover, inter-observer differences should be minor as the described thinking steps for QRS estimation using the hexaxial reference system follow objective criteria.

## Conclusion

**Fig. 2:**
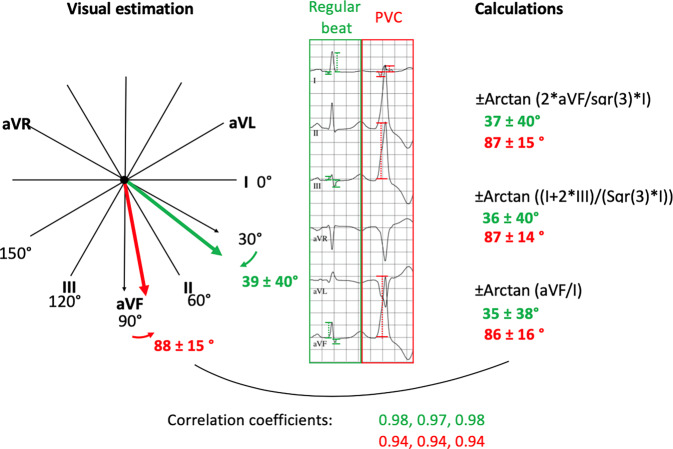
Summarizing figure. QRS axis determination of 216 QRS complexes of patients with outflow tract premature ventricular complexes (PVC) by visual estimation versus calculations

The authors confirm a strong correlation between calculated QRS axes and QRS axes estimated visually with the help of the hexaxial reference system to an accuracy of 10°, including when considering the axes of outflow tract PVCs. The estimation method can therefore be considered sufficient for most clinical and scientific questions (Fig. 2).

## Data Availability

The data sets can be requested from the head of the ethics committee of Hannover Medical School or from the corresponding author upon reasonable request.

## Declarations

The study complied with the Declaration of Helsinki and was approved by the local ethics committee. All participants provided written informed consent.
